# Tailored Mental Health Literacy Training Improves Mental Health Knowledge and Confidence among Canadian Farmers

**DOI:** 10.3390/ijerph17113807

**Published:** 2020-05-27

**Authors:** Briana N. M. Hagen, Sherilee L. Harper, Terri L. O’Sullivan, Andria Jones-Bitton

**Affiliations:** 1 Department of Population Medicine, University of Guelph, 50 Stone Road East, Guelph, ON N1G 2W1, Canada; sherilee.harper@ualberta.ca (S.L.H.); tosulliv@uoguelph.ca (T.L.O.); aqjones@uoguelph.ca (A.J.-B.); 2 School of Public Health, University of Alberta, 3-300 Edmonton Clinic Health Academy, 11405-87 Avenue, Edmonton, AB T7G 1C9, Canada

**Keywords:** mental health literacy, agriculture, evaluation, mental health, farmers

## Abstract

This study evaluated the impact of “In the Know” mental health literacy training for Canadian agriculture. We hypothesized that “In the Know” would significantly increase participants’ knowledge around mental health, confidence in recognizing mental health struggles, confidence in speaking about mental health with others, and confidence in helping someone who may be struggling with mental health. “In the Know” was a 4-h, in-person program delivered by a mental health professional who also had experience in agriculture. Six sessions were offered in Ontario, Canada in 2018. Participants were farmers and/or worked primarily with farmers. A pre-training paper questionnaire was administered, followed by a post-training questionnaire at the end of the session and 3 and 6 month post-training questionnaires via email. Wilcoxon signed-rank tests were performed to compare participants’ self-reported knowledge and confidence across four timepoints. “In the Know” significantly improved participants’ self-reported mental health knowledge and confidence in recognizing mental health struggles, speaking to others, and helping others who are struggling immediately following training and often at 3 and 6 months post-training. This is the first study among farming populations to measure program impact with 3- and 6-month follow-ups. Given the reported associations between mental health literacy and increased help-seeking, disseminating “In the Know” more broadly across farming communities may help to increase mental health literacy and thus increase help-seeking among farmers.

## 1. Introduction

Farmers across many nations, including the United States (US), Canada, the United Kingdom, and India, are reported to experience mental illness at higher rates than the general population and rural residents who do not farm [1,2,3,4,5]. A recent Canadian study reported that farmers had elevated levels of perceived stress, depression, anxiety, and burnout compared to general population norms [3]. Farmers are also less likely than non-farmers to seek help for their mental health [6]. Numerous studies have explored the relationship between barriers to help-seeking and mental health programming among farming populations [7,8,9]. The results of these studies have all discussed the relationship in which the individual farmer’s willingness to seek help from a program increases as the mental health service provider’s level of agricultural knowledge increases [7,8,9].

In Australia [10] and New Zealand [11], mental health literacy programming was developed specifically for farming populations and was successful in reaching farmers and increasing general mental health knowledge, improving attitudes towards those struggling with mental health, and increasing helping behaviours [11]. In 2011, the Australian Government and the University of Newcastle’s Centre for Rural and Remote Mental Health partnered to develop Farm-Link, a program which aimed to improve access to and the responsiveness of the available mental health services for farming populations [10]. An evaluation of this program found that a mental health service needed to be deemed credible by the farming community it intended to serve before community members would feel comfortable accessing that service [10]. In this instance, the farming community highlighted the importance of the mental health service providers having a strong background in and/or understanding of farming and the realities that farmers face day-to-day [10]. 

Other research has reported that the impact of the stigma that exists around help-seeking for mental health struggles may be exacerbated in rural populations [12]. An improvement in mental health literacy, which aims to increase knowledge and decrease stigma around mental health, can thus serve as a tool for improving access to mental health services [13,14]. 

Mental Health First Aid (MHFA) is a standardized mental health literacy training program that aims to teach participants to “approach, support, and refer” individuals who are experiencing a mental health crisis [15]. MHFA also aims to increase participants’ knowledge around mental disorders, adjust beliefs about people who may be struggling with their mental health, and influence behaviours that can enable help-seeking [15]. MHFA has been rigorously assessed for use with the general population, as well as some specific sub-populations including rural football club leaders and extension agents in rural communities, and is considered a gold standard mental health literacy training program [15]. Nevertheless, as MHFA was developed for the general population, it may have characteristics that limit uptake in farming populations specifically. In 2016, Jones–Bitton (unpublished) offered a session of MHFA to an agricultural-based audience in Ontario, Canada, and while the feedback received was positive overall, it was strongly suggested that MHFA may not be the ideal fit for Canadian farmers. Specifically, the length of the program (12 h over 2 days), the depth of the program materials, and the possible lack of agricultural knowledge amongst the individuals delivering the training were listed as barriers for the participating farmers. 

Evans, Craig, and Hodinott (2019) argued that interventions must be adapted to the cultural context to maximize their impact [16]. Farmers are a sub-population with a specific set of needs and potential barriers that must be addressed in order to create an effective mental health literacy (MHL) training program, including financial pressures, stigma, isolation, and limited access to services, along with a lack of willingness to utilize mental health services [10,11,17]. Additionally, Hawkins and colleagues (2017) developed a three-staged framework aimed at the co-production of public health interventions [18]. These stages, including (1) stakeholder consultation and evidence review, (2) co-production, and (3) prototyping, were reported as useful for ensuring that public health interventions (like mental health literacy training) were successful within the communities they aimed to serve [18]. Therefore, in collaboration with an agricultural stakeholder working group (consisting of approximately 30 people that met in person once or twice a year), “In the Know” MHL training for Canadian agriculture (“In the Know”) was developed following the stages reported within the Hawkins et al. framework for intervention co-production [18]. The “In the Know” training program was designed to specifically address the barriers identified by the agricultural community, including the length of the program and the agricultural knowledge of the trainer, and to ensure that the topics were explored through an agricultural lens.

Considering that this is the first mental health literacy program in Canada that was developed to address farmer-identified barriers to accessing mental health programming, the goal of this evaluation study was to assess the effectiveness of “In the Know”. The specific objectives were to determine if participants’ self-reported (1) knowledge of mental health struggles, (2) confidence in recognizing mental health struggles, (3) confidence in talking about mental health, and (4) confidence in helping others who may be struggling with their mental health improved immediately and 3 and 6 months post-training. We hypothesized that the participants who completed “In the Know” training would report significant improvements across all four objectives. We also hypothesized that increases in knowledge and confidence would remain significantly improved at both the 3-month and 6-month post-training follow-ups.

## 2. Materials and Methods

### 2.1. Study Design

In consultation with our agricultural working group, we developed a mental health literacy training program through an agricultural lens. The working group included individuals from several agricultural sectors, including farming, veterinary medicine, and government, along with members from social work, psychology, epidemiology, education, and mental health literacy trainers. Working with the group, we identified relevant mental health topics and developed learning objectives that were centred around increasing general mental health knowledge, confidence in speaking with others about mental health, and confidence in helping others who may be experiencing a mental health struggle. The working group provided guidance on the ideal length, delivery method/metric, and other characteristics of the training program to maximize uptake in the agricultural community.

#### 2.1.1. Training Program

“In the Know” was developed as a 4-h, in-person training program delivered by a trained mental health professional who had experience in the field of agriculture. Material was delivered as an interactive discussion with slides, videos, and handouts as visual aids. Vignettes, used as training tools for specific mental health outcomes, were developed and pre-tested by the working group to ensure that the example scenarios were accurate and realistic within a farming context. As determined by the working group, the training was designed to be delivered in small groups (*n* < 20 participants) to facilitate group discussion and learning. There was no monetary cost to attend the training, and a hot lunch was provided to participants.

A series of six evaluation sessions of “In the Know” across Ontario was conducted. To recruit participants, farm industry groups in the study regions were contacted via the working group and email invitations were sent to their electronic mailing lists. The first twenty individuals per region to respond to the invitation were registered for the evaluation session. Participants were required to be farmers and/or work primarily with farmers in their day-to-day roles. The sessions were held in Ontario, Canada (Guelph, Woodstock (x2), Niagara, Lennox-Addington, and Stratford) on weekdays, from 10:00 a.m.–2:00 p.m., between 23 October 2018 and 26 November 2018. The timing of the training and all logistical decisions were informed by the working group. This evaluation study had 97 total participants. Each session had between 14 and 19 participants, with a median group size of 16. 

#### 2.1.2. Questionnaires

Immediately prior to beginning the training session, participants were invited to complete a pre-training paper questionnaire (T0) on location. Participants were provided with a unique three digit “participant code” that was entered on all four surveys, to help ensure confidentiality and to allow for longitudinal analyses. Participants who provided informed consent were given a 38-item self-report questionnaire which used previously validated items [19] to assess the four areas of interest for this study: (1) participants’ current level of knowledge around general mental health concepts; (2) their confidence in recognizing mental health struggles; (3) their confidence in speaking to others about mental health; and (4) their confidence in helping people who were experiencing a mental health struggle (see supplemental information for full survey). Demographic information was also collected at T0. This questionnaire took approximately 15–20 min to complete. 

Immediately after the in-person training, participants were given a 19-item post-training questionnaire (T1) on location to re-assess the four areas of interest. Ten questions were identical to the T0 survey (except for demographic information), measuring the self-reported impact of “In the Know” with respect to the four areas of interest. Questions also asked for feedback on “In the Know” itself, including satisfaction with length, depth of content, and recommendations for improvement. The post-training questionnaires were the same at all three follow-up assessments. At three and six months post-training, questionnaires (each 10–15 min long) T2 and T3, respectively, were emailed to all participants and contained a link to an electronic version of the post-training questionnaire (Qualtrics© survey software). Reminders were sent at weekly intervals (for a maximum of three reminders) to participants who had not completed the follow-up questionnaires. This study methodology was approved by the University of Guelph Research Ethics Board (REB# 18-07-013).

### 2.2. Statistical Analyses

Data from the paper questionnaires (T0 and T1) were entered manually into STATA 15 © (StataCorp LLC, College Station, TX, USA) and validated for accuracy by a second research team member. The electronic survey data (T2 and T3) were uploaded to STATA 15 © for analysis. Descriptive statistics (e.g., frequencies and proportions) were used to summarize the data. Consistent with previous work in the area of mental health literacy among farmers, and as the data were non-parametric, differences in knowledge, confidence, and helping behaviours at T0, T1, T2, and T3 were assessed using the Wilcoxon signed-rank test [11,20]. A significant difference was denoted by a *p*-value of <0.05. 

## 3. Results

### 3.1. Participants

Of the 97 participants, 91 (93.8%) completed pre- (T0) and post-training (T1) paper questionnaires. The 3-month follow-up email questionnaire (T2) had an 80% response rate (*n* = 78) and the 6-month follow-up (T3) had a 77% response rate (*n* = 75). Demographic information for the participants is reported in Table 1. Over half of the participants were female, and the majority of participants were between 18 and 59 years of age. Farming represented the largest proportion of employment among the participants (37.4%).

### 3.2. Participants’ Self-Reported Data

#### 3.2.1. General Mental Health Knowledge 

**Figure 1:**
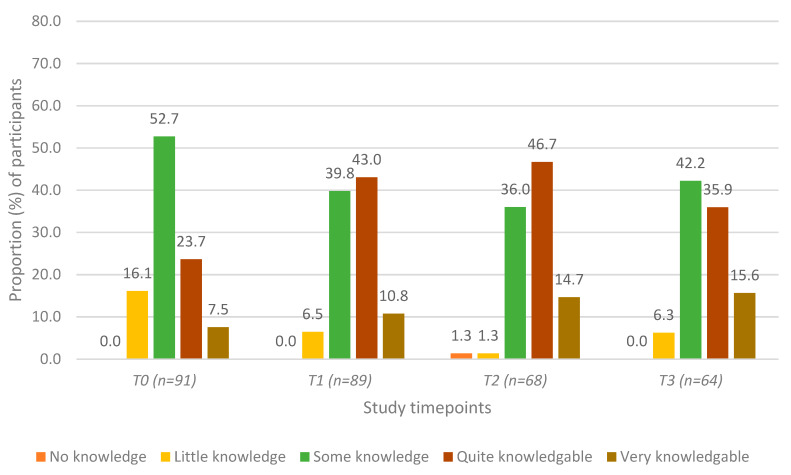
Self-reported level of general mental health knowledge among agricultural community members in Ontario, Canada at four timepoints: pre-training (T0), post-training (T1), 3 months post-training (T2), and 6 months post-training (T3).

Self-reported levels of general mental health knowledge at each of the study timepoints are presented in Figure 1. There was a statistically significant increase in participants’ self-reported general mental health knowledge between T0 and T1; this increase remained significant at T2 and T3 (Table 2, Row A). There were no significant changes in knowledge between T1, T2, and T3 (all *p* > 0.05). 

#### 3.2.2. Confidence in Recognizing Mental Health Struggles in Others

**Figure 2:**
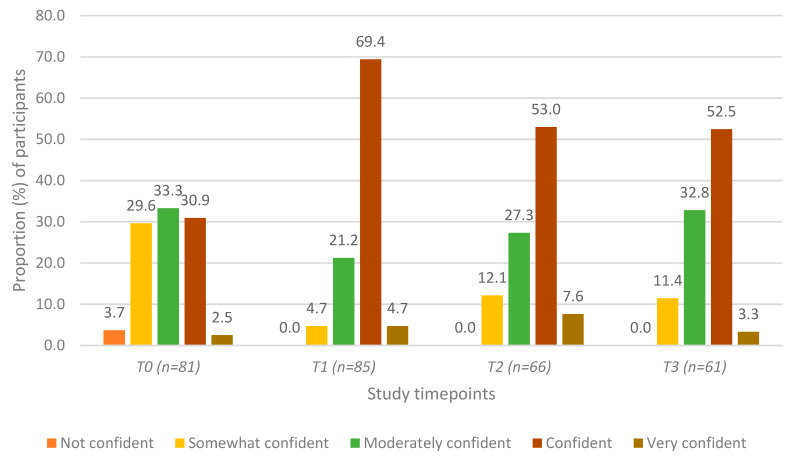
Self-assessment of confidence in recognizing signs and symptoms of mental health struggles among agricultural community members in Ontario, Canada at four timepoints: pre-training (T0), post-training (T1), 3 months post-training (T2), and 6 months post-training (T3).

Participants’ self-assessment of how confident they were in their ability to recognize the signs and symptoms of mental health struggles at each of the four timepoints is presented in Figure 2. The proportion of participants who “agreed” or “strongly agreed” that they were able to recognize signs and symptoms significantly increased three-fold (increasing from 29% to 68%) between T0 and T1, and this increase remained significant at the T2 and T3 assessments (Table 2, Row B). The proportion of participants who “agreed” or “strongly agreed” that they were confident decreased slightly from 63.8% to 52.0% between T1 and T2, although the difference was not statistically significant; there were no significant changes between T2 and T3 (Table 1, Row B).

#### 3.2.3. Confidence in Speaking to Others about Mental Health

**Figure 3:**
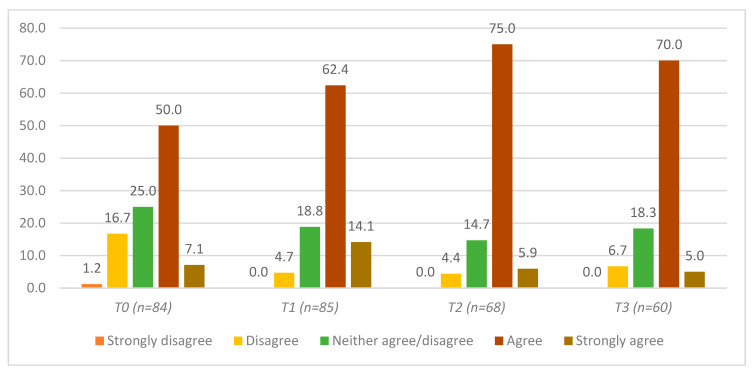
Self-assessment of confidence, if approached, in speaking to others about mental health in general (among agricultural community members in Ontario, Canada) at four timepoints: pre-training (T0), post-training (T1), 3 months post-training (T2), and 6 months post-training (T3).

Figure 3 shows that more than half of the participants (53.9%) indicated that they were comfortable speaking about mental health at T0. This proportion significantly increased between T0 and the T1 (74.4%), T2 (80%), and T3 (75%) timepoints (Table 2, Row C). There were no statistically significant differences in reported comfort in speaking about mental health between T1, T2, and T3 (Table 2, Row C).

#### 3.2.4. Confidence in Helping Others Who May Be Struggling with Their Mental Health

**Figure 4:**
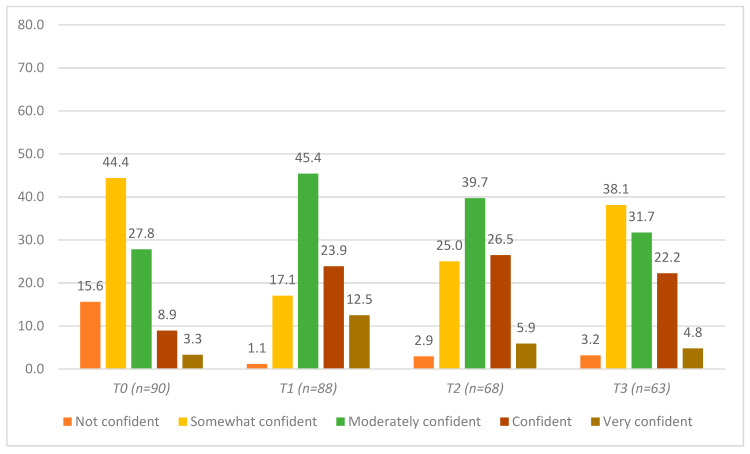
Self-assessment of confidence in helping others with a mental health struggle among agricultural community members in Ontario, Canada at four timepoints: pre-training (T0), post-training (T1), 3 months post-training (T2), and 6 months post-training (T3).

Figure 4 and Table 2, Row D illustrate participants’ self-reported confidence in helping others with mental health struggles. The proportion of participants who reported being “confident” or “very confident” significantly improved from approximately 12% at T0 to 36.4% at T1 and remained significantly increased at the T2 and T3 assessments (32.4% and 27%, respectively). When including those participants who felt “moderately” confident, this gap widened: approximately 40% of participants reported feeling moderately or more confident to help others at T0, while ~82% reported feeling moderately or more confident at T1 (Figure 4). Participants who reported feeling “not confident” decreased from 15.4% at T0 to 1.1% at T1, 2.9% at T2, and 3.2% at T3 (Figure 4).

### 3.3. Participant Feedback on the Content, Helpfulness, Relevance, and Usefulness of “In the Know”

When asked about the content of “In the Know” at T1, 89.9% (80/89) of participants indicated that the content level was “just right”. Four participants (4.5%) indicated that it was “a bit too much” and five participants (5.6%) indicated that it was “a bit too light”. Participants were asked about the usefulness of the training using a 5-point Likert scale format (from 1 = “extremely” to 5 = “not at all”). Most participants reported that “In the Know” was “extremely” or “very” helpful (93.3%; 83/89). The remaining six participants (6.7%) indicated that “In the Know” was “sort of” helpful.

Participants were asked to rate the usefulness, relevance, and realism of the vignettes used to exemplify the content in the training. For all three characteristics, 91.9% (79/86) of participants indicated they were “extremely” or “very” useful, relevant, and realistic. Three participants (3.4%) declined to answer the questions and the remainder rated them as neutral (7/89; 7.9%). In the open-text responses, participants discussed “In the Know” as “practical with useful examples that were relatable” and stated that the program allowed them to “have the knowledge base to talk about mental health in the context of the agricultural community”. 

Lastly, 100.0% (88/88) of the responding participants indicated that they would recommend “In the Know” training to others. In the open-ended responses, participants indicated that they valued the format of “In the Know”, specifically the delivery of the materials through an agricultural lens. One participant reported, “It is specific to ag [agriculture] and resonates with the audience. People feel connected and meaningful conversation was created.” Another participant reported, “The examples were great to help break down the stereotypes that farmers are one-man armies and don’t need ‘that type of help’.”

## 4. Discussion

Canadian farmers experience increased levels of adverse mental health outcomes, including stress, depression, anxiety, and burnout, compared to normative populations [3]. Evidence suggests that increasing mental health literacy can help to reduce mental health struggles within the general population [15]. Previous work in farming populations has indicated that tailoring MHL programs to farmers may result in the greatest impact for participants [10,11,16]. We evaluated the effectiveness of “In the Know”, a mental health literacy training program that was developed in partnership with the Canadian agricultural community and partners from mental health, over a 6-month period. To our knowledge, this is the only mental health literacy program of its kind in Canada and is the first study in farming populations to assess the effectiveness of tailored mental health literacy training over time (i.e., 6 months of follow-ups). The results of our study showed improvement on all four measures of interest, namely self-reported knowledge of mental health, confidence in recognizing the signs and symptoms of mental health struggles, confidence in speaking to others about mental health, and confidence in helping others with a mental health struggle. These improvements remained statistically significant at T1, T2, and T3. 

Confidence in recognizing the signs and symptoms of mental health struggles continued to increase from T0 between T1, T2, and T3. It is plausible that as an individual had time to practice the skills they had learned through “In the Know”, they became more confident in using those skills to recognize the signs of a mental health struggle over time. This result is encouraging when considering previous research that has reported that increased MHL helps to increase mental health help-seeking and decrease overall mental health struggles in communities [15]. Thus, farmers who complete “In the Know” training and continue to practice their skills may have the potential to positively impact their communities.

Our evaluation study builds on the formal evaluation of the MHL training that was tailored to the agricultural community in New Zealand (GoodYarn), which showed improvements in mental health awareness, confidence, and knowledge from pre- to post-training but indicated that future research would benefit from longer-term follow-ups [11]. The “In the Know” evaluation addressed this gap, assessing the impact of the training beyond the “immediate post-training timepoint” by including follow-up assessments at 3 and 6 months post-training. Our results provide evidence that the knowledge and skills gained through “In the Know”, and other mental health literacy training programs that have been tailored to the farming community, may remain effective over time.

“In the Know” was developed in response to both anecdotal and empirical evidence that mental health programming developed with an agricultural lens would make potential participants more comfortable and better able to relate to the material [15,18]. More specifically, “In the Know” builds on research conducted on the Farm-Link [10] and GoodYarn [11] programs, both of which have been successful in reaching farmers and increasing general mental health knowledge, attitudes towards those struggling with mental health, and helping behaviours [11]. Our own qualitative research similarly showed that farmers were more likely to attend a mental health literacy course like “In the Know” if the content was developed with agricultural examples and delivered by a facilitator that understood the lifestyle and stresses of farming (unpublished data). Our program was developed with farmers, using an agricultural lens, and this appears to have had a beneficial impact as 100% of our participants indicated that they would recommend “In the Know” training to other farmers. 

Similarly to GoodYarn, our program was developed in direct response to an identified program gap by farming communities who did not feel that MHFA fully met their needs in terms of program content and accessibility [11]. “In the Know” focused on increasing mental health literacy in a way that was accepted by the sample population, the agricultural community in Canada. This approach is consistent with the notion of general mental health promotion, whereby individual resilience is increased by fostering a supportive environment through programming [21].

Another goal of “In the Know” was to ensure that farmers themselves were trained in MHL. The Farm-Link program previously targeted frontline agricultural workers to participate in MHFA, creating “casefinders” in hopes of preparing them to help farmers who may be experiencing mental health struggles [10]. When consulting our stakeholders for the development of “In the Know”, as suggested in the stage one framework for intervention co-production and prototyping [18], they agreed that frontline agricultural workers (e.g., veterinarians, milk-truck drivers, seed sales representatives) required mental health literacy training. However, they firmly believed that farmers themselves would benefit from increased mental health literacy. When considering MHL training for farmers, our stakeholders identified the time commitment and cost of MHFA training to be potential barriers. “In the Know” was developed to address these identified gaps within MHFA, decreasing the time commitment from 12 h to 4 h, incorporating only the mental health outcomes that stakeholders identified as essential topics to address, using agricultural examples, and ensuring that the facilitators had agricultural backgrounds in addition to formal mental health training. 

### Limitations

As with several investigations of mental health literacy training, the evaluation of “In the Know” did not include a control group; therefore, we cannot compare the impact of the program with those who did not receive “In the Know” training. Given that MHFA is considered an industry gold standard for mental health literacy [22], a suitable next step would be to conduct an equivalency trial between “In the Know” and MHFA. This comparison would be particularly poignant given the Canadian agricultural community’s reflections that effective mental health literacy training within their community requires appropriate (agri)cultural context, which MHFA does not currently address.

While the questionnaire items were previously validated and widely used in the literature [19], they were self-reported and therefore could be prone to bias. Participants may have misinterpreted the questions themselves or over/underestimated their abilities, impacting the magnitude of the associations found within the study. Additionally, because our study sample was comprised of volunteers, it is possible that they may have been more inclined towards mental health literacy than randomly selected participants.

The overall response rate for this study decreased over the 6-month follow-up period. It is possible that the participants who completed the T2 and T3 assessments were those that were most impacted by the program and, therefore, more engaged, leading to a response bias whereby the reported scores for knowledge and confidence were overestimated. Regrettably, we do not have data with reasons for loss to follow-up. Within our study, follow-up was conducted via email, which resulted in some loss to follow-up due to participants changing jobs and therefore changing email addresses. One participant died during the follow-up period. However, the response rate at the 6-month follow-up remained over 75%, which is >20% higher (more than one standard deviation higher) than the most recently reported response-rate averages within the literature on public health interventions [23,24]. 

Direct comparisons between the effectiveness of “In the Know” and MHFA were not possible in this study. MHFA has a standardized questionnaire that is used for their assessments which is not directly comparable to the data from our study. In the future, an equivalency trial directly comparing the effectiveness of “In the Know” to that of MHFA would help to ensure “In the Know” is at least equal to MHFA in improving knowledge, confidence, and helping behaviours among farming populations.

## 5. Conclusions

In order to increase mental health literacy among Canadian farmers, “In the Know” was developed in partnership with the agricultural community to deliver MHL training through an agricultural lens. Within this evaluation study, “In the Know” was shown to significantly increase participants’ knowledge around mental health, confidence in recognizing mental health struggles, confidence in speaking about mental health with others, and confidence in helping someone who may be struggling with mental health, which are the primary goals of any MHL training. Investigating whether “In the Know” performs similarly to well-recognized and validated programs (e.g., MHFA) through an equivalency trial is a warranted next step for this research, as it would be helpful to know if a shorter program tailored to farmers is as beneficial as such “gold standard” programs. Practically, “In the Know” can teach farmers about mental health as well as increase their confidence in speaking about mental health and helping fellow farmers who may be experiencing mental health struggles. Given the reported associations between mental health literacy and increased help-seeking in the community, disseminating “In the Know” more broadly across farming communities may help to not only increase MHL but also decrease adverse mental health outcomes among farmers.

## Figures and Tables

**Table 1:** Demographic information (*n* = 91) for Canadian agricultural community members who participated in the “In the Know” evaluation sessions.

Participant Demographics	*n* (%)
**Gender:**	
Male	35 (38.5)
Female	52 (57.1)
Declined to answer	4 (4.4)
**Age:**	
18–29	16 (17.6)
30–39	28 (30.8)
40–49	18 (19.8)
50–59	22 (24.2)
60–69	5 (5.5)
70+	2 (2.2)
**Education:**	
High school	12 (13.0)
College diploma/university degree	54 (59.3)
Graduate degree	23 (25.3)
Declined to answer	2 (2.2)
**Agricultural Employment:**	
Farmer	34 (37.4)
Government extension agent	8 (8.8)
Veterinarian	4 (4.4)
Agronomist	6 (6.6)
Administrative support	4 (4.4)
Engineer	3 (3.3)
Manager/team leader	4 (4.4)
Graduate student/research assistant	3 (3.3)
Livestock/production specialist	6 (6.6)
Banker/insurance adjuster	4 (4.4)
Consultant	3 (3.3)
Other ^1^	8 (8.8)
Declined to answer	4 (4.4)

^1^ Other occupations included social worker (*n* = 1), self-employed (*n* = 1), sales representative (*n* = 1), communications (*n* = 1), environmental specialist (*n* = 2), and technology support (*n* = 2).

**Table 2:** Self-reported differences * for agricultural community members in Ontario, Canada in general mental health knowledge, confidence, and helping behaviours at pre-training (T0), post-training (T1), 3 months post-training (T2), and 6 months post-training (T3). Significant *p*-values are indicated in bold.

Outcome		Pre-Training (T0) (*n* = 97)	Post-Training (T1) (*n* = 97)	3 Months Post (T2) (*n* = 78)	6 Months Post (T3) (*n* = 75)
Row A. General mental health knowledge	Pre-training	-	**<0.0001**	**<0.0001**	**0.0003**
	Post-training	**<0.0001** ^1^	-	0.732	0.8589
	3 months post	**<0.0001**	0.732	-	0.4962
	6 months post	**0.0003**	0.8589	0.4962	-
Row B. Confidence recognizing mental health struggles	Pre-training	-	**<0.0001**	**<0.0001**	**<0.0001**
	Post-training	**<0.0001**	-	**0.0285**	**0.0065**
	3 months post	**<0.0001**	**0.0285**	-	0.9561
	6 months post	**<0.0001**	**0.0065**	0.9561	-
Row C. Comfort speaking to others about mental health	Pre-training	-	**<0.0001**	**0.0004**	0.0504
	Post-training	**<0.0001**	-	0.3743	0.1493
	3 months post	**0.0004**	0.3743	-	1.0000
	6 months post	0.0504	0.1493	1.0000	-
Row D. Comfort in helping others	Pre-training	-	**<0.0001**	**<0.0001**	**<0.0001**
	Post-training	**<0.0001**	-	**0.0203**	**<0.0001**
	3 months post	**<0.0001**	**0.0203**	-	0.6469
	6 months post	**<0.0001**	**<0.0001**	0.6469	-

* Wilcoxon signed-rank test. ^1^ Significant *p*-values are denoted in bold. Significance was defined as *p* < 0.05.

## Supplementary Materials

The supplementary materials are available online at https://www.mdpi.com/1660-4601/17/11/3807/s1.
