# Supplementary material for: Tailored Mental Health Literacy Training Improves Mental Health Knowledge and Confidence among Canadian Farmers
Source: Int J Environ Res Public Health. 2020 May 27;17(11):3807. doi: 10.3390/ijerph17113807 (PMC7313011; doi:10.3390/ijerph17113807)
Supplement: Supplementary file 1 [file ijerph-17-03807-s001.pdf]

UNIVERSITY of GUELPH

ONTARIO VETERINARY COLLEGE
Department of Population Medicine

Mental Health Literacy Training – PRE-Training Questionnaire

1. To create a unique, confidential survey code, please put the last 3 letters of your last name and the last 3 digits of your phone number. This will be used for this survey, and the subsequent post-training survey. This code will help to ensure your identity is kept confidential.

____

2. Have you already participated in any mental health training? For example, Mental Health First Aid, SafeTalk, or ASIST?

- Yes
- No

3. How would you rate your **current knowledge** about the following mental health conditions?

a) General mental health conditions (e.g. depression, anxiety, inability to concentrate)

- I have no knowledge about these conditions
- I have little knowledge about these conditions
- I have some knowledge about these conditions
- I am quite knowledgeable about these conditions
- I am very knowledgeable about these conditions
- Decline to answer

b) Violence or aggressive behaviour associated with mental health issues

- I have no knowledge about this
- I have little knowledge about this
- I have some knowledge about this
- I am quite knowledgeable about this
- I am very knowledgeable about this
- Decline to answer

c) Sexual problems associated with mental health issues

- I have no knowledge about this
- I have little knowledge about this
- I have some knowledge about this
- I am quite knowledgeable about this
- I am very knowledgeable about this
- Decline to answer

d) Relationship difficulties associated with mental health issues

- I have no knowledge about this
- I have little knowledge about this
- I have some knowledge about this
- I am quite knowledgeable about this
- I am very knowledgeable about this
- Decline to answer

e) Depression with suicidal thoughts

- I have no knowledge about this
- I have little knowledge about this
- I have some knowledge about this
- I am quite knowledgeable about this
- I am very knowledgeable about this
- Decline to answer

f) Suicide ideation or attempt

- I have no knowledge about this
- I have little knowledge about this
- I have some knowledge about this
- I am quite knowledgeable about this
- I am very knowledgeable about this
- Decline to answer

g) Substance abuse (e.g. alcohol, drugs)

- I have no knowledge about this
- I have little knowledge about this
- I have some knowledge about this
- I am quite knowledgeable about this
- I am very knowledgeable about this
- Decline to answer

4. How **confident** would you feel in helping someone with a mental health condition?

- Not at all confident
- Somewhat confident
- Moderately confident
- Confident
- Very confident
- Decline to answer

5. To the best of your knowledge, in the last six months (i.e. since April), have you had contact with anyone with a mental health condition?

- Yes
- No [SKIP to QUESTION 9]

6. How many people with a mental health issue have you had contact with in the last six months?

____

7. Did you provide any help to the last person you encountered that was experiencing a mental health issue?

- No [GO TO 8A]
- I provided a little help [GO TO 8B]
- I provided some help [GO TO 8B]
- I provided a lot of help [GO TO 8B]
- I was the primary support [GO TO 8B]

8A. [IF NO TO 7] For what reasons did you not provide any help?

____

____

____

____

8B. [IF YES TO 7] Please briefly describe the type of help you provided, including any recommendations that you may have made.

____

____

____

____

9. Please indicate your level of agreement with the following statements:

a) "I am confident that I could recognize when someone is struggling with their mental health/wellness."

- Strongly agree
- Agree
- Neither agree nor disagree
- Disagree
- Strongly disagree
- Don't know
- Decline to answer

b) “I am comfortable speaking to others about their mental health.”

- Strongly agree
- Agree
- Neither agree nor disagree
- Disagree
- Strongly disagree
- Don’t know
- Decline to answer

c) “I am comfortable speaking to others about their possible substance abuse.”

- Strongly agree
- Agree
- Neither agree nor disagree
- Disagree
- Strongly disagree
- Don’t know
- Decline to answer

d) “If someone approached me to have a conversation about their mental health, I am confident I would know how to respond in an appropriate way.”

- Strongly agree
- Agree
- Neither agree nor disagree
- Disagree
- Strongly disagree
- Don’t know
- Decline to answer

e) “If someone approached me to have a conversation about their substance abuse, I am confident I would know how to respond in an appropriate way.”

- Strongly agree
- Agree
- Neither agree nor disagree
- Disagree
- Strongly disagree
- Don’t know
- Decline to answer

f) “I believe that farmers with <u>depression</u> could snap out of it if they wanted.”

- Strongly agree
- Agree
- Neither agree nor disagree
- Disagree
- Strongly disagree
- Don’t know

- Decline to answer

g) "I believe that farmers with depression with suicidal thoughts could snap out of it if they wanted."

- Strongly agree
- Agree
- Neither agree nor disagree
- Disagree
- Strongly disagree
- Don't know
- Decline to answer

h) "I believe that farmers who say they are stressed are complaining too much."

- Strongly agree
- Agree
- Neither agree nor disagree
- Disagree
- Strongly disagree
- Don't know
- Decline to answer

10. Now we would like you to tell us what you think most other people believe. Please indicate your level of agreement with each of the following statements:

a) "Most people believe that farmers with depression could snap out of it if they wanted."

- Strongly agree
- Agree
- Neither agree nor disagree
- Disagree
- Strongly disagree
- Don't know
- Decline to answer

b) "Most people believe that farmers with depression with suicidal thoughts could snap out of it if they wanted."

- Strongly agree
- Agree
- Neither agree nor disagree
- Disagree
- Strongly disagree
- Don't know
- Decline to answer

c) “Most people believe that farmers who say they are stressed are complaining too much.”

- Strongly agree
- Agree
- Neither agree nor disagree
- Disagree
- Strongly disagree
- Don’t know
- Decline to answer

11. How willing would you be to **move next door** to someone with each of the following mental health conditions?

a) Depression

- Definitely would not be willing
- Probably would not be willing
- Unsure
- Probably would be willing
- Definitely would be willing
- Decline to answer

b) Depression with suicidal thoughts

- Definitely would not be willing
- Probably would not be willing
- Unsure
- Probably would be willing
- Definitely would be willing
- Decline to answer

c) Substance use/abuse

- Definitely would not be willing
- Probably would not be willing
- Unsure
- Probably would be willing
- Definitely would be willing
- Decline to answer

12. We encourage you to provide any explanation for your answers here:

____

____

____

____

13. How willing would you be to **spend an evening socializing** with someone with each of the following mental health conditions?

a) Depression
- Definitely would not be willing
- Probably would not be willing
- Unsure
- Probably would be willing
- Definitely would be willing
- Decline to answer

b) Depression with suicidal thoughts
- Definitely would not be willing
- Probably would not be willing
- Unsure
- Probably would be willing
- Definitely would be willing
- Decline to answer

c) Substance use/abuse
- Definitely would not be willing
- Probably would not be willing
- Unsure
- Probably would be willing
- Definitely would be willing
- Decline to answer

d) Any other mental illness
- Definitely would not be willing
- Probably would not be willing
- Unsure
- Probably would be willing
- Definitely would be willing
- Decline to answer

14. We encourage you to provide any explanation for your answers here:

____

____

____

____

15. How willing would you be to **make friends** with someone with each of the following mental health conditions?

a) Depression
- Definitely would not be willing
- Probably would not be willing
- Unsure
- Probably would be willing
- Definitely would be willing
- Decline to answer

b) Depression with suicidal thoughts
- Definitely would not be willing
- Probably would not be willing
- Unsure
- Probably would be willing
- Definitely would be willing
- Decline to answer

c) Substance use/abuse
- Definitely would not be willing
- Probably would not be willing
- Unsure
- Probably would be willing
- Definitely would be willing
- Decline to answer

d) Any other mental illness
- Definitely would not be willing
- Probably would not be willing
- Unsure
- Probably would be willing
- Definitely would be willing
- Decline to answer

16. We encourage you to provide any explanation for your answers here:

____

____

____

____

17. How willing would you be to have someone with each of the following mental health conditions **work closely with you on a job**?

a) Depression
- Definitely would not be willing
- Probably would not be willing
- Unsure
- Probably would be willing
- Definitely would be willing
- Decline to answer

b) Depression with suicidal thoughts
- Definitely would not be willing
- Probably would not be willing
- Unsure
- Probably would be willing
- Definitely would be willing
- Decline to answer

c) Substance use/abuse
- Definitely would not be willing
- Probably would not be willing
- Unsure
- Probably would be willing
- Definitely would be willing
- Decline to answer

d) Any other mental illness
- Definitely would not be willing
- Probably would not be willing
- Unsure
- Probably would be willing
- Definitely would be willing
- Decline to answer

18. We encourage you to provide any explanation for your answers here:

____

____

____

____

19. How willing would you be to have someone with each of the following mental health conditions **marry into your family**?

a) Depression
- Definitely would not be willing
- Probably would not be willing
- Unsure
- Probably would be willing
- Definitely would be willing
- Decline to answer

b) Depression with suicidal thoughts
- Definitely would not be willing
- Probably would not be willing
- Unsure
- Probably would be willing
- Definitely would be willing
- Decline to answer

c) Substance use/abuse
- Definitely would not be willing
- Probably would not be willing
- Unsure
- Probably would be willing
- Definitely would be willing
- Decline to answer

d) Any other mental illness
- Definitely would not be willing
- Probably would not be willing
- Unsure
- Probably would be willing
- Definitely would be willing
- Decline to answer

20. We encourage you to provide any explanation for your answers here:

____

____

____

____

21. In your own words, please describe what “mental health” means to you:

____

____

____

The next few questions are demographic questions that will help us compare our study sample to the general population. Please remember that the survey is **confidential**, and all data will kept confidential.

22. What is your gender?

____

23. What is your occupation?

____

24. What is your age in years?
- 18-29
- 30-39
- 40-49
- 50-59
- 60-69
- 70 or older
- Decline to answer

25. What is your relationship status?
- Single and not in a committed dating relationship
- In a committed dating relationship but not living together
- Living common-law
- Married
- Separated
- Divorced
- Widowed
- Decline to answer

26. What is your highest level of education received?
- Grade school
- High school
- College / trade / technical school
- University – undergraduate studies

- University – graduate studies
- Decline to answer

27. Do you belong to the following groups?

a) Person with a mental health issue
- Yes
- No
- Decline to answer

b) Care for a person with a mental health issue
- Yes
- No
- Decline to answer

28. Have you yourself <u>ever</u> experienced a mental health issue?
- Yes
- No
- Decline to answer

29. Has anyone in your family or circle of close friends <u>ever</u> experienced a mental health issue?
- Yes
- No
- Decline to answer

This next set of questions are taken from the standardized Medical Outcomes Study Short-Form Health Survey (SF-12). They ask for your views about your health, how you feel, and how well you are able to do your usual activities on a typical day. Please remember that your responses are **confidential**, and all data will be kept strictly confidential.

30. In general, would you say your health is:
- Excellent
- Very good
- Good
- Fair
- Poor
- Decline to answer

31. The following questions are about activities you might do during a typical day. To what extent does <u>your health now limit you</u> in these activities?

a) **Moderate activities**, such as moving a table, pushing a vacuum cleaner, bowling, or playing golf?
- Yes – limited a lot
- Yes – limited a little
- No – not limited at all
- Decline to answer

b) Climbing **several** flights of stairs?

- Yes – limited a lot
- Yes – limited a little
- No – not limited at all
- Decline to answer

32. During the past 4 weeks, (i.e. since September 23) have you had any of the following problems with your work or other regular activities as a result of your physical health:

a) Have you **accomplished less** than you would like as a result of your physical health?

- Yes
- No
- Decline to answer

b) Were you limited in the **kind** of work or other activities as a result of your physical health?

- Yes
- No
- Decline to answer

33. During the past 4 weeks, have you had any of the following problems with your work or other regular daily activities as a result of your mental health and/or emotional problems (such as feeling depressed or anxious):

a) Have you **accomplished less** than you would like as a result of your mental health and/or emotional problems?

- Yes
- No
- Decline to answer

b) Did you not do work or other activities as **carefully** as usual as a result of your mental health and/or emotional problems?

- Yes
- No
- Decline to answer

34. During the past 4 weeks, how much did pain interfere with your normal work (including both work outside the home and housework)?

- Not at all
- A little bit
- Moderately
- Quite a bit
- Extremely
- Decline to answer

The last few questions are about how you feel and how things have been with you during the past 4 weeks (i.e. since September 23rd.) For each question, please give the one answer that comes closest to the way you have been feeling.

35. How much of the time during the past 4 weeks have you felt calm and peaceful?
- All of the time
- Most of the time
- A good bit of the time
- Some of the time
- A little of the time
- None of the time
- Decline to answer

36. How much of the time during the past 4 weeks did you have a lot of energy?
- All of the time
- Most of the time
- A good bit of the time
- Some of the time
- A little of the time
- None of the time
- Decline to answer

37. How much of the time during the past 4 weeks have you felt down?
- All of the time
- Most of the time
- A good bit of the time
- Some of the time
- A little of the time
- None of the time
- Decline to answer

38. How much of the time during the past 4 weeks has your physical health or mental health interfered with your social activities (like visiting with friends, relatives, etc.)?
- All of the time
- Most of the time
- A good bit of the time
- Some of the time
- A little of the time
- None of the time
- Decline to answer

That was our last question. Thank you for taking the time to complete this survey – your responses are very important to us.

If there is anything else you would like to share, please feel free to do so here:

____

____

____

____

____

____

____

____

ONTARIO VETERINARY COLLEGE
Department of Population Medicine

In the Know – POST-Training Questionnaire

PART ONE:

1. To create a unique, confidential survey code, please put the last 3 letters of your last name and the last 3 digits of your phone number. This will be used for this survey, and the subsequent post-training survey. This code will help to ensure your identity is kept confidential.

____

2. Did the '"In the Know": Building our knowledge & capacity around mental health' program influence the way you define mental health?
   A. Yes
   B. No
   C. Decline to answer

Please briefly explain:
____

____

____

____

3. Generally, how the content level of the session?
   1. Way too much
   2. A bit too much
   3. Just right
   4. A bit light
   5. Way too light

4. How was the pace of the session?
   1. Much too fast
   2. A bit too fast
   3. Just right
   4. A bit too slow
   5. Much too slow

5. The example vignettes (“stories”) were:

A. Realistic:

1	2	3	4	5
(Extremely realistic)				(Not realistic at all)

B. Relevant:

1	2	3	4	5
(Extremely relevant)				(Not relevant at all)

C. Useful:

1	2	3	4	5
(Extremely useful)				(Not useful at all)

6. Overall, I found this training to be:
   1. Extremely helpful
   2. Very helpful
   3. Sort of helpful
   4. Not very helpful
   5. Extremely unhelpful

7. Please write 3 words to describe this training:

_______________ _______________ _______________

8. Would you recommend ‘“In the Know”: Building our knowledge & capacity around mental health’ program to others in agriculture?
   A. Yes
   B. No
   C. Decline to answer

Please briefly explain:

____

____

____

____

9. What recommendations do you have for improving “In the Know” for Canadian agriculture?

____

____

____

If you have any additional comments, please feel free to share them here:

____

____

____

____

PART TWO:

1. How would you rate your **current knowledge** about the following mental health conditions?

a) General mental health conditions (e.g. depression, anxiety, inability to concentrate)
- I have no knowledge about these conditions
- I have little knowledge about these conditions
- I have some knowledge about these conditions
- I am quite knowledgeable about these conditions
- I am very knowledgeable about these conditions
- Decline to answer

b) Violence or aggressive behaviour associated with mental health issues
- I have no knowledge about these conditions
- I have little knowledge about these conditions
- I have some knowledge about these conditions
- I am quite knowledgeable about these conditions
- I am very knowledgeable about these conditions
- Decline to answer

c) Sexual problems associated with mental health issues
- I have no knowledge about this
- I have little knowledge about this
- I have some knowledge about this
- I am quite knowledgeable about this
- I am very knowledgeable about this
- Decline to answer

d) Relationship difficulties associated with mental health issues

- I have no knowledge about this
- I have little knowledge about this
- I have some knowledge about this
- I am quite knowledgeable about this
- I am very knowledgeable about this
- Decline to answer

e) Depression with suicidal thoughts

- I have no knowledge about this
- I have little knowledge about this
- I have some knowledge about this
- I am quite knowledgeable about this
- I am very knowledgeable about this
- Decline to answer

f) Suicide ideation or attempt

- I have no knowledge about this
- I have little knowledge about this
- I have some knowledge about this
- I am quite knowledgeable about this
- I am very knowledgeable about this
- Decline to answer

g) Substance abuse (e.g. alcohol, drugs)

- I have no knowledge about this
- I have little knowledge about this
- I have some knowledge about this
- I am quite knowledgeable about this
- I am very knowledgeable about this
- Decline to answer

2. How **confident** would you feel in helping someone with a mental health condition?

- Not at all confident
- Somewhat confident
- Moderately confident
- Confident
- Very confident
- Decline to answer

3. To the best of your knowledge, <u>in the last six months</u> (i.e. since April), have you had contact with anyone with a mental health condition?

- Yes
- No [SKIP to QUESTION 7]

4. How many people with a mental health issue have you had contact with in the last six months?

____

5. Did you provide any help to the last person you encountered that was experiencing a mental health issue?

- No [GO TO 6A]
- I provided a little help [GO TO 6B]
- I provided some help [GO TO 6B]
- I provided a lot of help [GO TO 6B]
- I was the primary support [GO TO 6B]

6A. [IF NO TO 5] For what reasons did you not provide any help?

____

____

____

____

6B. [IF YES TO 5] Please briefly describe the type of help you provided, including any recommendations that you may have made.

____

____

____

____

7. Please indicate your level of agreement with the following statements:

a) "I am confident that I could recognize when someone is struggling with their mental health/wellness."

- Strongly agree
- Agree
- Neither agree nor disagree
- Disagree
- Strongly disagree
- Don't know
- Decline to answer

b) “I am comfortable speaking to others about their mental health.”

- Strongly agree
- Agree
- Neither agree nor disagree
- Disagree
- Strongly disagree
- Don’t know
- Decline to answer

c) “I am comfortable speaking to others about their possible substance abuse.”

- Strongly agree
- Agree
- Neither agree nor disagree
- Disagree
- Strongly disagree
- Don’t know
- Decline to answer

d) “If someone approached me to have a conversation about their mental health, I am confident I would know how to respond in an appropriate way.”

- Strongly agree
- Agree
- Neither agree nor disagree
- Disagree
- Strongly disagree
- Don’t know
- Decline to answer

e) “If someone approached me to have a conversation about their substance abuse, I am confident I would know how to respond in an appropriate way.”

- Strongly agree
- Agree
- Neither agree nor disagree
- Disagree
- Strongly disagree
- Don’t know
- Decline to answer

f) “I believe that farmers with depression could snap out of it if they wanted.”

- Strongly agree
- Agree
- Neither agree nor disagree
- Disagree
- Strongly disagree
- Don’t know

- Decline to answer

g) “I believe that farmers with <u>depression with suicidal thoughts</u> could snap out of it if they wanted.”

- Strongly agree
- Agree
- Neither agree nor disagree
- Disagree
- Strongly disagree
- Don’t know
- Decline to answer

h) “I believe that farmers who say they are stressed are complaining too much.”

- Strongly agree
- Agree
- Neither agree nor disagree
- Disagree
- Strongly disagree
- Don’t know
- Decline to answer

8. How willing would you be to **move next door** to someone with each of the following mental health conditions?

a) Depression

- Definitely would not be willing
- Probably would not be willing
- Unsure
- Probably would be willing
- Definitely would be willing
- Decline to answer

b) Depression with suicidal thoughts

- Definitely would not be willing
- Probably would not be willing
- Unsure
- Probably would be willing
- Definitely would be willing
- Decline to answer

c) Substance use/abuse

- Definitely would not be willing
- Probably would not be willing
- Unsure
- Probably would be willing
- Definitely would be willing
- Decline to answer

9. We encourage you to provide any explanation for your answers here:

____

____

____

10. How willing would you be to **spend an evening socializing** with someone with each of the following mental health conditions?

a) Depression
- Definitely would not be willing
- Probably would not be willing
- Unsure
- Probably would be willing
- Definitely would be willing
- Decline to answer

b) Depression with suicidal thoughts
- Definitely would not be willing
- Probably would not be willing
- Unsure
- Probably would be willing
- Definitely would be willing
- Decline to answer

c) Substance use/abuse
- Definitely would not be willing
- Probably would not be willing
- Unsure
- Probably would be willing
- Definitely would be willing
- Decline to answer

d) Any other mental illness
- Definitely would not be willing
- Probably would not be willing
- Unsure
- Probably would be willing
- Definitely would be willing
- Decline to answer

11. We encourage you to provide any explanation for your answers here:

____

____

____

____

12. How willing would you be to **make friends** with someone with each of the following mental health conditions?

a) Depression

- Definitely would not be willing
- Probably would not be willing
- Unsure
- Probably would be willing
- Definitely would be willing
- Decline to answer

b) Depression with suicidal thoughts

- Definitely would not be willing
- Probably would not be willing
- Unsure
- Probably would be willing
- Definitely would be willing
- Decline to answer

c) Substance use/abuse

- Definitely would not be willing
- Probably would not be willing
- Unsure
- Probably would be willing
- Definitely would be willing
- Decline to answer

d) Any other mental illness

- Definitely would not be willing
- Probably would not be willing
- Unsure
- Probably would be willing
- Definitely would be willing
- Decline to answer

13. We encourage you to provide any explanation for your answers here:

____

____

____

____

15. How willing would you be to have someone with each of the following mental health conditions **work closely with you on a job**?

a) Depression
- Definitely would not be willing
- Probably would not be willing
- Unsure
- Probably would be willing
- Definitely would be willing
- Decline to answer

b) Depression with suicidal thoughts
- Definitely would not be willing
- Probably would not be willing
- Unsure
- Probably would be willing
- Definitely would be willing
- Decline to answer

c) Substance use/abuse
- Definitely would not be willing
- Probably would not be willing
- Unsure
- Probably would be willing
- Definitely would be willing
- Decline to answer

d) Any other mental illness
- Definitely would not be willing
- Probably would not be willing
- Unsure
- Probably would be willing
- Definitely would be willing
- Decline to answer

16. We encourage you to provide any explanation for your answers here:

____

____

____

____

17. How willing would you be to have someone with each of the following mental health conditions **marry into your family**?

a) Depression
- Definitely would not be willing
- Probably would not be willing
- Unsure
- Probably would be willing
- Definitely would be willing
- Decline to answer

b) Depression with suicidal thoughts
- Definitely would not be willing
- Probably would not be willing
- Unsure
- Probably would be willing
- Definitely would be willing
- Decline to answer

c) Substance use/abuse
- Definitely would not be willing
- Probably would not be willing
- Unsure
- Probably would be willing
- Definitely would be willing
- Decline to answer

d) Any other mental illness
- Definitely would not be willing
- Probably would not be willing
- Unsure
- Probably would be willing
- Definitely would be willing
- Decline to answer

18. We encourage you to provide any explanation for your answers here:

____

____

____

____

19. In your own words, please describe what "mental health" means to you:

____

____

____

____

That was our last question. Thank you for taking the time to complete this survey – your responses are very important to us.

If there is anything else you would like to share, please feel free to do so here:

____

____

____

____

____
